# Environmental tobacco smoke exposure during pregnancy affects complications and birth outcomes in women with and without asthma

**DOI:** 10.1186/s12884-020-03000-z

**Published:** 2020-05-20

**Authors:** Nasrin Fazel, Michael Kundi, Asghar Kazemzadeh, Habibollah Esmaily, Roya Akbarzadeh, Raheleh Ahmadi

**Affiliations:** 1 Sabzevar University of Medical Sciences, Sabzevar, Iran; 2 Center for Public Health, Medical University Vienna, Kinderspitalgasse 15, 1090 Vienna, Austria; 3 Department of Internal Medicine, Sabzevar University of Medical Sciences, Sabzevar, Iran; 4 Department of Biostatistics & Epidemiology, Neonatal Research Center, Mashhad University of Medical Sciences, Mashad, Iran; 5 Department of Anesthesia & Operating Room, College of Paramedics, Sabzevar University of Medical Sciences, Sabzevar, Iran; 6 Department of Obstetrics and Gynecology, Mobini Hospital, Sabzevar University of Medical Sciences, Sabzevar, Iran

**Keywords:** Asthma, Smoking, Environmental tobacco smoke, Pregnancy outcome

## Abstract

**Background:**

It is known that environmental tobacco smoke (ETS) has adverse effects on pregnancy and birth outcomes. We aimed to assess the impact of ETS in pregnant women with and without asthma.

**Methods:**

A cohort study was conducted from August 2014 to June 2015 enrolling 1603 pregnant women during their 2nd trimester. Data on tobacco exposure were collected at first visit and women were followed through pregnancy till postpartum.

**Results:**

Of the 1603 women, 231 reported passive smoking, 223 non-asthmatics and 8 asthmatics. Women exposed to ETS during pregnancy were more likely to have an infant admitted to the pediatric ward (10.8% vs. 6.5%, *p* = 0.026) and to have low one- and five-minute Apgar scores (1 min: 6.1% vs. 2.6%, *p* = 0.011; 5 min: 2.2% vs. 0.7%, *p* = 0.039). Complications of pregnancy were also elevated in women exposed to ETS (53.7% vs. 42.3%, *p* = 0.002). Asthma had no additional effect beyond the impact of ETS except for cesarean sections that were more frequent in women with asthma exposed to ETS.

**Conclusions:**

Due to the small number of women with asthma exposed to ETS, combined effects of asthma and ETS were only found for cesarean sections. Still counseling of pregnant women about adverse effects of ETS should consider women’s asthma as an additional reason to avoid ETS.

## Background

Passive Smoking is a major public health problem worldwide [1]. This could especially be the case during vulnerable periods like pregnancy [2]. Therefore, it is important to improve understanding of the association between maternal and child health outcomes and maternal smoke exposure across various social groups [3].

Exposure of nonsmoking pregnant women to environmental tobacco smoke (ETS) is associated with a number of adverse perinatal outcomes including lower birthweight, smaller head circumference and stillbirth. There is overall consistency in the literature about the negative effects of fetal and postnatal exposure to parental tobacco smoking on several outcomes: preterm birth, fetal growth restriction, low birth weight, sudden infant death syndrome, neurodevelopmental and behavioral problems, obesity, hypertension, type 2 diabetes, impaired lung function, asthma and wheezing [2, 4, 5]. This information is important for women, their families and healthcare providers, and reinforces the continued need for education on prevention of exposure to passive smoke. Smoke-free legislation in England was associated with clinically important reductions in severe adverse perinatal outcomes. It was associated with a 7.8% (95%CI 3.5–11.8; *p* < 0.001) reduction in stillbirths, a 3.9% (95%CI 2.6–5.1; p < 0.001) reduction in low birth weight, and a 7.6% (95%CI 3.4–11.7; *p* = 0.001) reduction in neonatal mortality [6]. However, prevalence of domestic ETS exposure and maternal smoking during pregnancy still remain high [7]. Although, globally, the proportion of women who smoked during pregnancy was low, in some countries very high fractions were found and overall about half of smoking women continued smoking daily in pregnancy (52.9%; 95% CI 45.6–60.3 [8];). Using biomarkers for ETS exposure it has been found that a substantial proportion of newborns are exposed [9].

It has been recommended that childhood asthma prevention programs should include smoking cessation strategies targeted towards smokers who live in the homes of smoking and nonsmoking pregnant women [10].

ETS operates as a cofactor with other insults such as recurrent infections to trigger wheezing, rather than as a factor that induces asthma, whereas in utero exposure increases physician-diagnosed asthma in the child [11–13].

Currently, available evidence supports the need to plan population health policies aimed at implementing educational programs to minimize tobacco smoke exposure during pregnancy and lactation [11].

There are substantial differences between regions with respect to women’s smoking and also smoking during pregnancy and similar differences apply to ETS [8, 14]. Therefore we undertook to study ETS and its potential impact on pregnancy and perinatal outcomes in pregnant women with and without asthma in Iran, a region with comparatively low smoking prevalence in women [15]. The disparities in active tobacco use and ETS exposure among race/ethnic groups underscore the importance of culturally and ethnically relevant factors that should be considered in interventions and surveillance in order to advance progress towards the goal of reducing tobacco’s harm.

Hence, the purposes of this study were to examine the association between self-reported passive smoke exposure during pregnancy and pregnancy complications and outcomes, and to assess whether this association is affected by women’s asthma.

## Methods

### Study subjects

This prospective study was conducted from August 2014 to April 2015 at the Mobini Hospital, Iran. All pregnant women in the 2nd trimester were eligible if they could be interviewed in Farsi. Additional inclusion criteria were: Capable of providing informed consent, good overall health without history of chronic disease other than asthma. Exclusion criteria were: Pregnancy complication in the first trimester, evidence of malignancy within the past 5 years, respiratory tract infections within 6 weeks preceding the evaluation. Overall, 1607 pregnant women during their second-trimester prenatal checkups (> 12 weeks of pregnancy) were screened for our study. At enrollment, women were interviewed by trained research assistants; follow-up interviews were conducted by phone at 20 (if first visit was before week 17), 28, and 36 weeks (±5 days) of gestation and in the hospital postpartum. In the follow-up interviews, information about changes in asthma symptoms, household and workplace conditions including active and passive smoking was collected. Overall, 1603 subjects, all of whom gave written informed consent, answered a questionnaire including information about active and passive smoking and their birth outcome was eventually evaluated by assessing delivery records. The study was approved by the Research Ethics Committee of Iran (Medsab Rec.93.36).

### Questionnaire

The questionnaire contained sections about demographic, medical, and pregnancy characteristics and active and passive smoking history. Household members’ cigarette smoke during pregnancy was assessed using previously applied questions [16, 17]. Additional questions were about characteristics of participants including age, place of living, ethnicity, education, previous pregnancies and births, medical history focusing on asthma and allergies. It contained questions about asthma symptoms, previous diagnosis, duration of asthma, current treatment, known allergies and allergic symptoms. Questions about active smoking were about daily tobacco consumption and duration of smoking and were based on previous studies. Passive smoking (ETS) was defined as occurring when a woman was living with someone who smokes at home or working together with someone who smokes at the workplace [7, 18, 19]. These data were used to allocate pregnant women to the following categories reflecting tobacco smoke exposure during pregnancy: i) no tobacco smoke exposure, ii) maternal active smoking at any point during pregnancy and iii) ETS exposure. Additional information including maternal weight gain during pregnancy, complications of current pregnancy and previous pregnancies was collected from clinical records.

The questions about smoking included: (1) Are you currently smoking? (2) Does your husband or partner smoke in your home? (3) Not including yourself or your husband or partner, does anyone else smoke cigarettes inside your home? (4) Do you spend time either at home, at your workplace or any other place where you are exposed to tobacco smoke? (see Supplementary Material 1).

In addition, the Asthma Control Questionnaire was applied [20].

Content validity of the questionnaire was evaluated by a professional board of six specialists in nursing and midwifery, health education, and smoking cessation. The questionnaire was administered by well-trained interviewers.

### Outcome variables

The outcome variables considered were complications of pregnancy (vaginal bleeding, urinary tract infection (UTI), vomiting/emesis, pre-eclampsia, premature rupture of membranes (PROM), gestational diabetes mellitus (GDM), cerclage, having pain three weeks before delivery and other complications) and delivery and birth outcome (gestational age at delivery, method of delivery, Apgar scores after 1 and 5 min, birth weight, admittance of newborn to the ICU), birth anomalies (e.g., anencephaly, urethral stenosis, omphalocele, spina bifida) and developmental anomalies (e.g. pathological reflexes).

### Statistical analysis

In a pilot study 200 women were enrolled and asthma prevalence was determined at 5%. This figure was used to estimate the sample size necessary for determining differences in risk of complications between women with and without asthma providing a power of 80% for an odds ratio (OR) of 2. The sample size was set to 1600 women, which is about 10% of all pregnant women in the city of Sabzevar in Iran in the period between August 2014 and April 2015. After this sample size was reached it turned out, however, that asthma prevalence was much lower (2%), therefore, we conducted a power analysis to determine if the study is still sufficiently powered to provide information on substantially increased relative risks. For attributes with a background frequency exceeding 15%, the power under the given conditions exceeds 80% for ORs of 3 or larger. Furthermore, we tested which effect size would be afforded to detect a combined effect of asthma and ETS assuming a prevalence of 14% for ETS. Under the same conditions as mentioned above, the study could detect an about 5-fold difference in ORs with 80% power applying the method proposed by Nam [21].

In the following, continuous data are summarized as mean ± standard deviation, categorical data as counts and percentages. Groups with and without ETS were compared by Fisher’s exact probability test (Fisher-Freeman-Halton test, if more than two categories) for categorical data, continuous data were compared by Student’s t tests. Multiple logistic regression analysis was applied using the Generalized Linear Model to test the single and combined effect of ETS and asthma controlling for age, active smoking, education and parity. *p* values below 0.05 were considered significant. All analyses were performed using SPSS 23, (IBM Corp., NY, USA).

## Results

Overall, 1607 pregnant women were screened during their second-trimester prenatal checkups (> 12 weeks of pregnancy). Four subjects were excluded: two did not consent, one had a spontaneous abortion during the 2nd trimester and could not be followed up, and one did not permit blood samples to be drawn from her infant, which was required for another part of the study.

Among the 1603 participants 231 (14.4%) reported exposure to ETS; of these women 8 (3.5%) were diagnosed with asthma. Among asthmatic women 3 (8.8%) were current smokers and 20 (1.3%) among non-asthmatic women were current smokers (*p* = 0.01). Except for education no difference in demographic characteristics were found between groups of women with and without ETS exposure (Table 1). Women reporting ETS had lower educational status (*p* < 0.001) and were also more likely to be active smokers (*p* < 0.001).

**Table 1:** Characteristics of pregnant women by environmental tobacco smoke (ETS). *p*-values from Fisher’s exact probability test

		ETS		
Characteristic	Category	No (*n* = 1372)	Yes (*n* = 231)	*p*-value
Age (years)	< 25 y	473 (34.5%)	85 (36.8%)	0.296
	25–29 y	424 (30.9%)	72 (31.2%)	
	30–34 y	268 (19.5%)	46 (19.9%)	
	35+ y	207 (15.1%)	28 (12.1%)	
Active smoking	yes	8 (0.6%)	15 (6.5%)	< 0.001
Residence	city	942 (68.7%)	148 (64.1%)	0.171
	village	430 (31.3%)	83 (35.9%)	
Ethnicity	Farsi	209 (15.2%)	29 (12.6%)	0.551
	Turk	1143 (83.3%)	198 (85.7%)	
	other	20 (1.5%)	4 (1.7%)	
Education	Elementary school	279 (20.3%)	67 (29.0%)	< 0.001
	High school	795 (57.9%)	141 (61.0%)	
	College/university	298 (21.7%)	23 (10.0%)	
Body weight	underweight (BMI < 18.5)	70 (5.1%)	12 (5.2%)	0.904
	normal (BMI 18.5- < 25)	1158 (84.4%)	197 (85.3%)	
	overweight (BMI > =25)	144 (10.5%)	22 (9.5%)	
Parity	1	553 (40.3%)	95 (41.1%)	0.486
	2	520 (37.9%)	77 (33.3%)	
	3	237 (17.3%)	46 (19.9%)	
	4+	62 (4.5%)	13 (5.6%)	
Asthma	yes	26 (1.9%)	8 (3.5%)	0.137

Overall in 704 women (43.9%) complications of pregnancy were recorded. Women exposed to ETS had a significantly higher prevalence of such complications (53.7% vs. 42.3%, *p* = 0.002) but without any specific complication being particularly elevated. Asthma did not add significantly to the prevalence if occurring in combination with ETS (Tables 2 and 3).

**Table 2:** Complications of pregnancy by environmental tobacco smoke (ETS). Odds ratio (OR) for ETS and 95% confidence intervals (CI) unadjusted and adjusted for age, active smoking, education and parity. *p*-values from General Linear Model

	ETS		OR (95% CI)		ETS	Asthma^a^
	No (n = 1372)	Yes (n = 231)	crude	adjusted	*p*-value	*p*-value
Any Complication	580 (42.3%)	124 (53.7%)	1.58 (1.20–2.09)	1.57 (1.18–2.09)	0.002	0.361
Vaginal bleeding	121 (8.8%)	25 (10.8%)	1.25 (0.80–1.98)	1.28 (0.80–2.07)	0.304	0.313
UTI	126 (9.2%)	29 (12.6%)	1.42 (0.92–2.18)	1.30 (0.83–2.03)	0.247	0.584
Vomiting	274 (20.0%)	57 (24.7%)	1.31 (0.95–1.82)	1.32 (0.94–1.85)	0.115	0.836
Cerclage	11 (0.8%)	1 (0.4%)	0.54 (0.07–4.19)	0.57 (0.05–6.50)	0.633	1.000
PE	65 (4.7%)	13 (5.6%)	1.20 (0.65–2.21)	1.22 (0.64–2.31)	0.542	0.260
Pain ≥3 wk. bef. Delivery	43 (3.1%)	12 (5.2%)	1.69 (0.88–3.26)	1.80 (0.93–3.49)	0.083	0.968
PROM	60 (4.4%)	10 (4.3%)	0.99 (0.50–1.96)	1.04 (0.51–2.10)	0.916	0.984
GDM	24 (1.7%)	9 (3.9%)	2.28 (1.04–4.96)	2.13 (0.94–4.83)	0.052	0.882
Other	14 (1.0%)	1 (0.4%)	0.42 (0.06–3.22)	0.47 (0.06–3.62)	0.467	0.999

*UTI* urinary tract infection, *PE* preeclampsia, *PROM* premature rupture of membranes, *GDM* gestational diabetes mellitus

^a^*p*-value for interaction effect with ETS

**Table 3:** Complications of pregnancy and pregnancy outcomes in women with (w/) and without (w/o) asthma by environmental tobacco smoke (*p*-values for interaction see Tables 2 and 4)

		w/o asthma (*n* = 1569)		w/ asthma (*n* = 34)	
		ETS		ETS	
Parameter	Category	No (*n* = 1346)	Yes (*n* = 223)	No (*n* = 26)	Yes (*n* = 8)
Gestational age at delivery (weeks)	preterm (< 34)	23 (1.7%)	8 (3.6%)		
	late preterm (34- < 37)	74 (5.5%)	16 (7.2%)		
	early term (37- < 39)	293 (21.8%)	41 (18.4%)	6 (23.1%)	2 (25.0%)
	full term (39- < 41)	795 (59.1%)	125 (56.1%)	15 (57.7%)	6 (75.0%)
	late term (41+)	161 (12.0%)	33 (14.8%)	5 (19.2%)	0 (0.0%)
Cesarean section	yes	460 (34.2%)	76 (34.1%)	11 (42.3%)	7 (87.5%)
Apgar 1 min	7–10	1311 (97.4%)	209 (93.7%)	26 (100.0%)	8 (100.0%)
	< 7	35 (2.6%)	14 (6.3%)		
Apgar 5 min	7–10	1337 (99.3%)	218 (97.8%)	26 (100.0%)	8 (100.0%)
	< 7	9 (0.7%)	5 (2.2%)		
Admittance of newborn	yes	86 (6.4%)	24 (10.8%)	3 (11.5%)	1 (12.5%)
Anomaly	yes	5 (0.4%)	2 (0.9%)	1 (3.8%)	0 (0.0%)
Any complication	yes	566 (42.1%)	121 (54.3%)	14 (53.8%)	3 (37.5%)
Vaginal bleeding	yes	114 (8.5%)	24 (10.8%)	7 (26.9%)	1 (12.5%)
UTI	yes	124 (9.2%)	27 (12.1%)	2 (7.7%)	2 (25.0%)
Vomiting	yes	269 (20.0%)	56 (25.1%)	5 (19.2%)	1 (12.5%)
Cerclage	yes	11 (0.8%)	1 (0.4%)		
PE	yes	64 (4.8%)	11 (4.9%)	1 (3.8%)	2 (25.0%)
Pain ≥3 wk. bef. Delivery	yes	42 (3.1%)	12 (5.4%)	1 (3.8%)	0 (0.0%)
PROM	yes	59 (4.4%)	10 (4.5%)	1 (3.8%)	0 (0.0%)
GDM	yes	22 (1.6%)	8 (3.6%)	2 (7.7%)	1 (12.5%)
Other	yes	12 (0.9%)	1 (0.4%)	2 (7.7%)	0 (0.0%)
Birth weight (g)	mean ± SD	3153 ± 457	3108 ± 507	3146 ± 464	3333 ± 491

Gestational age at delivery did not differ between women with and without exposure to ETS (with ETS exposure 38.8 ± 2.2 weeks vs. without 38.9 ± 1.8 weeks). Also method of delivery was not different with slightly over one third with cesarean section in both groups. There was no significant relationship of ETS with birth weight of the child (w/ ETS: 3110 ± 504 g vs. w/o ETS: 3156 ± 458 g, *p* = 0.169). Apgar score, both after 1 and 5 min, was significantly more often below 7 in women exposed to ETS and the percentage of children needing admission to the pediatric unit was significantly higher in those born to mothers exposed to ETS (10.8% vs. 6.5%, *p* = 0.011) (Table 4). Asthma in combination with ETS did not show a interaction effect except for method of delivery with asthmatic women exposed to ETS showing a significantly higher proportion with cesarean section (7/8 women with asthma and ETS exposure; *p* = 0.032).

**Table 4:** Characteristics of pregnancy and pregnancy outcomes by environmental tobacco smoke (ETS). Odds ratio (OR) for ETS and 95% confidence intervals (CI) unadjusted and adjusted for age, active smoking, education and parity. *p*-values from General Linear Model

		ETS		OR (95% CI)		ETS	Asthma^a^
		No (n = 1372)	Yes (n = 231)	crude	adjusted	*p*-value	*p*-value
Gestational age at delivery (weeks)	preterm (< 34)	23 (1.7%)	8 (3.5%)	2.15 (0.94–4.91)	2.26 (0.98–5.22)	0.210	0.459
	late preterm (34- < 37)	74 (5.4%)	16 (6.9%)	1.34 (0.76–2.37)	1.37 (0.76–2.45)		
	early term (37- < 39)	299 (21.8%)	43 (18.6%)	0.89 (0.61–1.29)	0.90 (0.61–1.31)		
	full term (39- < 41)	810 (59.0%)	131 (56.7%)	1^#^	1^#^		
	late term (41+)	166 (12.1%)	33 (14.3%)	1.23 (0.81–1.86)	1.19 (0.77–1.84)		
Method of delivery	Vaginal delivery	901 (65.7%)	148 (64.1%)	1^#^	1^#^		
	Cesarean	471 (34.3%)	83 (35.9%)	1.07 (0.80–1.44)	1.23 (0.90–1.66)	0.191	0.032
Apgar 1 min	7–10	1337 (97.4%)	217 (93.9%)	1^#^	1^#^		
	< 7	35 (2.6%)	14 (6.1%)	2.46 (1.30–4.66)	2.68 (1.40–5.11)	0.003	1.000
Apgar 5 min	7–10	1363 (99.3%)	226 (97.8%)	1^#^	1^#^		
	< 7	9 (0.7%)	5 (2.2%)	3.35 (1.11–10.09)	3.55 (1.16–10.88)	0.026	1.000
Birth weight (g)	mean ± SD	3156 ± 458	3110 ± 504	1.28 (0.79–2.08)	1.23 (0.91–1.66)	0.169	0.161
Admittance of newborn	yes	89 (6.5%)	25 (10.8%)	1.75 (1.10–2.79)	1.87 (1.16–3.01)	0.011	0.541
Anomaly	yes	6 (0.4%)	2 (0.9%)	1.99 (0.40–9.91)	1.26 (0.21–7.67)	0.800	0.113

^#^reference category

^a^*p*-value for interaction effect with ETS

## Discussion

We found that Apgar scores and the rate of transfer of the newborn to a pediatric ward or neonatal intensive care unit (NICU) were significantly affected by ETS. The causes of admission to NICU were asphyxia, low Apgar and low blood sugar. Because we found no association of ETS with birth weight these results indicate that the developmental maturity of the newborn was affected without an impact in fetal growth.

Second-hand smoke exposure is now recognized as an important cause of adult and child morbidity and mortality. Pregnancy is an ideal opportunity to intervene with mothers and families to prevent and control tobacco use, and should be a priority for both tobacco control and maternal and child health care [22]. Our results are consistent with earlier studies on tobacco smoke exposure [19, 23, 24]. The fraction of active smokers (1.4%) was too low in our study to allow a specific analysis, but other studies demonstrate that active smoking is related to the same endpoints and only the impact seems to be more pronounced [25, 26]. It has been shown that cigarette or hookah smoking during pregnancy increases serum levels of thyroid hormones T3 and T4, which may explain fetal weight loss and subsequent low birth weight [27]. The number of active smokers among the pregnant women in our sample was too low to asses an influence on these outcomes, however, also passive smoking did not affect birth weight while other studies reported decreased birth weight and an increase in small-for-gestational-age infants from passive maternal smoking [28]. However, there was no clear dose-response relationship. Two studies [25, 29] demonstrated a sharp decline in birth weight at a certain level of ETS exposure. It is possible that this level was not exceeded in most of the women in our ETS exposed group. It has been hypothesized that the influence of ETS on the neonate observed in some studies could be due to the volatile organic compounds present in side-stream smoke [30] that may affect development of the fetus. This effect may depend on the conditions of the indoor environment and on climate. In the area of our study temperature rarely falls below zero °C and average temperature is above 20 °C already in April, thus natural window ventilation maybe sufficient to rapidly remove side-stream smoke.

Complications of pregnancy were more prevalent among women exposed to ETS but no single complication was significantly elevated. This is due to the relationship between power of the statistical test and the absolute frequency of the endpoint studies. Since having any complication is more frequent than having a specific complication, lack of an association for specific complications can be attributed to too low frequencies. While no specific complication was significantly increased, larger differences were noted for vomiting (24.7% vs. 20.0%) and gestational diabetes mellitus (3.9% vs. 1.7%). To our knowledge, no study has previously described such effects. While active smoking is associated with many adverse effects including preterm labor, premature rupture of membranes, and placental abruption [13, 23, 31] no such effect were apparent for ETS in our study.

We found a statistically significant association between maternal education and ETS. Already in 1992 Martinez, Cline [17] noted that mothers with 12 or less years of formal education were twice more likely to be current smokers than mothers with higher education. Tobacco use during pregnancy was reported in all WHO regions, but some countries had much higher maternal smoking rates [22], such as Nepal (5.9%), Jordan (9.6%), and Turkey (15.0%) than found in our cohort (1.4%).

Neither for complications during pregnancy nor for birth outcomes had asthma a substantial interaction effect with ETS. It seems, therefore, that woman with asthma and their offspring are not less or more at risk for experiencing adverse effects from ETS. The only exception was the rate of cesarean sections that were more than twice as frequent in women with asthma exposed to ETS than in those without asthma. Women with asthma had more frequently cesarean sections (53% vs. 34%) and this frequency increased even more in those exposed to ETS (87.5%). This is consistent with a large data-base of pregnancies that revealed an increased frequency of cesarean sections in women with asthma [32]. The observed further increase in women exposed to ETS points to increased respiratory problems during delivery in these women. This is consistent with the assumption of an irritation of the airways due to passive smoking and the increased likelihood of bronchoconstriction due to asthma.

Hodyl et al. reported that maternal asthma and cigarette smoking during pregnancy are both independently associated with adverse perinatal outcomes and, combined, increase the risk for urinary tract infections [33]. Also in our study risk of urinary tract infections was elevated, however, the increase did not reach statistical significance. We also found no significantly increased risks from ETS for pain ≥3 week before delivery, bleeding, preeclampsia, or premature rupture of membranes. Although there are biological explanations for a relationship of active and passive maternal smoking with spontaneous onset of labor and some complications of pregnancy, such as placental vasoconstriction and increased levels of catecholamines, both able to initiate labor; disruptions of the integrity of the amniotic membranes, interference with protein metabolism and maternal immunity leading to increased risk of infection, none of the investigated outcomes has universally been recognized as associated with passive smoking.

Passive smoking significantly increases the risk of an episode of uncontrolled asthma during pregnancy, which is likely to have adverse effects on pregnancy outcome. However, further evidence is needed regarding the effect of active and passive tobacco exposure during pregnancy on asthma control, also for the purpose of being able to provide the best possible advice to pregnant women with asthma exposed to tobacco smoke.

There are some limitations of our study that should be noted. Although the study was planned to have sufficient power to study asthma in combination with ETS, it turned out that the prevalence of asthma was less than half the figure expected from a pilot study. Therefore, the effect size from an interaction effect that can be detected in our study was large and more subtle effects could have been missed. Furthermore we relied on self-reported active and passive smoking. Substantiating self-reports by objective measurements such as breathing tests or cotinine was not covered by the ethics vote. In many circumstances and especially in countries where tobacco smoking is still widespread, like in Iran (but mostly among men), assessment by trained personnel can be considered reliable [25, 34]. We were not able to quantify the exposure, such as the number of hours a day exposed to ETS and the degree of exposure (such as ventilation efficiency), and so were not able to assess a possible dose–response relationship. There is also the possibility of response bias, since pregnant women might be hesitant to admit being exposed to ETS. We assessed a large number of endpoints and avoided to correct for multiplicity to not overlook a possible relationship. Therefore, some of our findings should be addressed in further trials. Despite these limitations, there has been no other study that recruited participants from the 12^th^week of gestation and followed them until delivery. The prospective nature of this study is its main strength. Since standard methods of assessment of complications of pregnancy and delivery have been applied, the results appear to be generalizable to all pregnant women.

## Conclusions

This study indicates that women exposed to tobacco smoke during pregnancy were more likely to have an infant admitted to the pediatric ward and to have low one- and five-minute Apgar scores. Complications of pregnancy were also elevated in women exposed to ETS but without specific complications being particularly affected. Asthma had no additional effect on the impact of ETS except for cesarean sections that were very frequent in women with asthma that were exposed to ETS. Childhood asthma prevention programs should include smoking cessation strategies targeted towards smokers who live in the homes of smoking and nonsmoking pregnant women. Public health policies should be oriented not only towards smoking cessation of pregnant women, but also reinforce elimination of ETS exposure.

## Supplementary information

**Additional file 1.**

## Data Availability

The datasets used and analyzed during the current study are available from the corresponding author on reasonable request.

## Abbreviations

BMI: Body mass index
CI: Confidence interval
ETS: Environmental tobacco smoke
GDM: Gestational diabetes mellitus
ICU: Intensive care unit
NICU: Neonatal intensive care unit
OR: Odds ratio
PE: Preeclampsia
PROM: Premature rupture of membranes
UTI: Urinary tract infection
WHO: World Health Organization
