# Supplementary material for: Environmental tobacco smoke exposure during pregnancy affects complications and birth outcomes in women with and without asthma
Source: BMC Pregnancy Childbirth. 2020 May 20;20:314. doi: 10.1186/s12884-020-03000-z (PMC7240917; doi:10.1186/s12884-020-03000-z)
Supplement: Supplementary file 1 — Additional file 1. [file 12884_2020_3000_MOESM1_ESM.docx]

# Supplementary Material 1

## Interview Guide

To be completed by interviewer

INTRODUCTION:

Good morning/good afternoon, my name is____________, I work in the field of public health at the Medical University Vienna. We are conducting a study related to asthma in pregnant women attending a Medical Education Center of the city of Sabzevar. This study will help us to better understand the factors promoting this disease among pregnant women and will provide recommendations for additional health programs. Your answers will be treated as confidential information and will be used only for the purposes of this study; therefore, your total honesty in your replies will be extremely valuable to our purposes. This survey will take around 30 minutes to complete. We appreciate your time and responses. If you consent, you will be contacted again two or three times during your pregnancy for a short update and your medical records will be used. Furthermore, after delivery we may ask you to provide a blood sample and to allow us to use a routine blood sample from your baby for testing of allergens. Do you agree to participate?

A. General

Code assigned to the survey:

Code assigned to the interviewer:

Date of interview: dd/mm/yyyy

Pregnant woman informed consent: No-0 Yes-1

Is the participant eligible for the study? No-0 Yes-1

INSTRUCTIONS:

Please listen very carefully to each question before you answer it.

Select the answer that best describes what you know, believe or feel is the right one.

Select only one answer for each question.

(For written part and participants that are able to read). When selecting your answer, please draw a circle around the number located at the right side. If you want to change your answer, you can erase it by clearly striking it out!)

B. Sociodemographic Information

1. How old are you?

□ Under 25 □ 26-40 □ 41-55 □ 56 or older

2. What is the highest level of education you have achieved?

□illiterate □ Grammar school

□ High school or equivalent vocational/technical school (2 years)

□ Some college □ Bachelor's degree □ Master's degree □Doctoral degree

□ Other

3. Occupation □ Employed □other

4. What is your current marital status? □ Married □ Divorced □ Separated □ Co-habitant □ Other

5. To what ethnic group do you belong? □Turk □ Fars □Other □ I do not want to answer

B. History of asthma

First, we would like to ask a few questions about you and the time before you got pregnant with your new baby

1. Have you had a sudden episode or recurrent episodes of severe coughing, whistling sounds when breathing or shortness of breath? □Yes □ No
2. Have you had colds that affected your chest or take more than 10 days to recover? □Yes □ No
3. Have you had episodes of coughing, wheezing or shortness of breath during a particular season or time of the year? □Yes □ No
4. Have you had episodes of coughing, wheezing or shortness of breath at certain places or when exposed to certain things (e.g. animals, tobacco smoke, perfumes)? □Yes □ No
5. Have you had episodes of coughing, wheezing or shortness of breath at night that awakened you in the early morning? □Yes □ No
6. Have you ever had episodes of coughing, wheezing or shortness of breath after running or moderate exercise or other physical activity? □Yes □
7. Have you used any medication that helped you breathing? □Yes □ No
8. Were your symptoms relieved when the medications were used? □Yes □ No

C. Smoking, Alcohol, Supplementation

1. Are you currently smoking? □Yes □ No
2. Have you smoked cigarettes in the past 2 two years? □Yes □ No
3. Does your husband or partner smoke inside your home? □Yes □ No
4. Not including yourself or your husband or partner, does anyone else smoke cigarettes inside your home? □Yes □ No
5. Do you spend time either at home, at your workplace or any other place where you are exposed to tobacco smoke? □Yes □ No
6. Have you had any alcoholic drinks in the past two years? □Yes □ No
7. During the last three months, how many times a week did you take a multivitamin, a prenatal vitamin, or a folic acid vitamin?
   □ not at all □ 1 to 3 times a week □ 4 to 6 times a week □ every day of the week

D. Maternal medical history

1. During the 3 months before you got pregnant with your new baby, did you have any of the following health problems?
2. Asthma □Yes □ No
3. High blood pressure (hypertension) □Yes □ No
4. Anemia (poor blood, low iron) □Yes □ No
5. Heart problems □Yes □ No
6. Epilepsy (seizures) □Yes □ No
7. Thyroid problems □Yes □ No
8. Depression □Yes □ No
9. Anxiety □Yes □ No
10. Autoimmune diseases □Yes □ No
11. Hepatitis □Yes □ No
12. Did you have any of the following problems during your most recent pregnancy?
13. Vaginal bleeding □Yes □ No
14. Kidney or bladder (urinary tract) infection □Yes □ No
15. Severe nausea, vomiting, or dehydration □Yes □ No
16. Cervix had to be sewn (cerclage) □Yes □ No
17. High blood pressure, hypertension (including pregnancy-induced hypertension), preeclampsia, or toxemia □Yes □ No
18. Problems with the placenta (such as abruptio placentae or placenta previa) □Yes □ No
19. Labor pains more than 3 weeks before my baby was due (preterm or early labor) □Yes □ No
20. Water broke more than 3 weeks before my baby was due (premature rupture of membranes) □Yes □ No
21. What infections or diseases were you told to have?

□Genital warts (HPV) □Herpes □ Chlamydia □ Gonorrhea □Pelvic inflammatory disease (PID) □Syphilis □Group B Strep (Beta Strep) bacterial vaginitis □ Trichomoniasis (Trich) □ Yeast infections □Urinary tract infection (UTI) □Other

1. Do any of your close family members who are related to you by blood (mother, father, sisters, or brothers) have had any of the conditions listed below?
   1. Diabetes □No □Yes □ Don’t Know
   2. Heart problems □No □ Yes □ Don’t Know
   3. High blood pressure (hypertension) □No □ Yes □ Don’t Know
   4. Depression □No □ Yes □ Don’t Know
   5. Postpartum depression □No □ Yes □ Don’t Know
   6. Asthma □No □ Yes □ Don’t Know
